# Toxic Effects of Nonylphenol on Neonatal Testicular Development in Mouse Organ Culture

**DOI:** 10.3390/ijms21103491

**Published:** 2020-05-15

**Authors:** Hyun-Jung Park, Mingtian Zhang, Won-Young Lee, Kwon-Ho Hong, Jeong Tae Do, Chankyu Park, Hyuk Song

**Affiliations:** 1 Department of Stem Cell and Regenerative Biology, Konkuk University, 1 Hwayang-dong, Gwangjin-gu, Seoul 05029, Korea; sunrisep@konkuk.ac.kr (H.-J.P.); zmt8856@naver.com (M.Z.); hongk@konkuk.ac.k (K.-H.H.); dojt@konkuk.ac.kr (J.T.D.); chankyu@konkuk.ac.kr (C.P.); 2 Department of Beef Science, Korea National College of Agricultures and Fisheries, Jeonju-si, Jeonbuk 54874, Korea; leewy81@korea.kr

**Keywords:** nonylphenol, testis, meiosis, germ cell

## Abstract

Nonylphenol (NP) is an alkylphenol that is widely used in chemical manufacturing. Exposure to this toxic environmental contaminant has been shown to negatively affect the reproductive system. Herein, we evaluated the toxicity of NP in mouse testes, while using in vitro organ culture. Mouse testicular fragments (MTFs), derived from five-day postpartum neonatal mouse testes, were exposed to different concentrations of NP (1–50 μM) for 30 days. The results showed that NP impaired germ cell development and maintenance. Furthermore, NP significantly downregulated the transcript levels of both undifferentiated and differentiated germ cell marker genes relative to those in controls. In particular, a high dose of NP (50 µM) led to complete germ cell depletion and resulted in spermatogenic failure, despite the presence of Sertoli and Leydig cells. In addition, the mRNA expression of steroidogenic enzymes, such as steroidogenic acute regulatory protein (STAR), Cytochrome P450 Family 11 Subfamily A Member 1 (Cyp11α1), Cytochrome P450 17A1 (Cyp17α1), and androgen receptor (AR), increased with increasing concentration of NP. Conversely, the expression of estrogen receptor alpha (ESR1) and Cytochrome P450 family 19 subfamily A member 1 (Cyp19α1) in NP-exposed MTFs decreased when compared to that of the control. Taken together, this study demonstrates that NP has a negative effect on prepubertal spermatogenesis and germ cell maintenance and it disrupts steroidogenesis and induces hormonal imbalance in MTFs.

## 1. Introduction

Nonylphenol (NP) is an alkylphenol that is widely used in the manufacture of antioxidants, lubricating oil additives, textiles, and agriculture products, such as pesticides, emulsifiers, and dispersants [1]. NP is an endocrine disruptor that is capable of interfering with the normal functioning of hormones in various organisms [2,3]. It has been detected in nearly all water matrices in the environment, including ground- and drinking water. Furthermore, it enters the food chain via contaminated foods, such as vegetables, milk, and meat [4,5]. Hence, toxicological research on NP has increased in recent years.

Human plasma samples that were obtained from healthy volunteers were found to contain 0.2–0.3 ng/mL of NP [6]. The concentration of NP in human blood samples varies widely, ranging from below the detection limit up to 53.21 ng/g [7,8,9]. Human urine samples were found to contain NP concentrations between 2.77 and 42.06 ng/mL [10]. In addition, studies in Japan and Italy reported the presence of NP in human breast milk in the range of 0.65–1.4 ng/g [11] and 13.4–56.3 ng/mL, respectively [12]. However, it is unclear how NP affects the human body, as data regarding the effects of NP exposure on human cells are limited. Nevertheless, studies have shown that NP disrupts the decidualization of endometrial stromal cells, thereby exerting reproductive toxicity in women [13]. It has also been shown to induce apoptosis and DNA damage in human keratocytes [14].

NP is also an endocrine disruptor and it causes reproductive health problems, such as infertility. Several studies have shown that NP can cause a significant decrease in sperm motility and viability in livestock animals [15,16]. In addition, it negatively affects spermatogenic development as well as sperm count and motility in rodents [17,18]. Further, NP leads to serious testicular dysfunction in rats, as revealed by reduced testis size, and it induces cell death via oxidative stress in Sertoli cells [19,20]. Similarly, NP disturbs the testicular structure and suppresses spermatogenesis in fish [21]. In five-week-old male rats, exposure to NP led to spermatogenic degeneration and pronounced deficits in epididymal sperm count, motility, and function. In another study, high doses of NP (60 mg/kg/2 d) impaired testicular development and function by reducing cell proliferation and inducing apoptosis, involving oxidative stress-related p53-Bcl-2/Bax and Fas/FasL pathways [22].

Recent studies have used the testis organ culture method in rodents for assessing toxicity to the reproductive system [23,24,25]. In our previous studies, we successfully developed adult-like testes from neonatal testes and evaluated the toxicity of resmethrin in neonatal testes in organ culture [26]. Similarly, Zhang et al. investigated the effects of bisphenol A (BPA) and diethylhexyl phthalate (DEHP) on neonatal testis in an in vitro organ culture [27]. Lopes et al. studied the effects of 7-ethyl-10-hydroxycamptothecin (SN38) on the testes and ovaries of prepubertal mice [28]. The advantage of this method is that it eliminates the requirement for experimental animal models, and thus prevents suffering.

Herein, neonatal testicular tissue that was developed using the organ culture method was treated with NP, followed by an evaluation of early testes development. For the first time, we were able to estimate the toxicity of NP in developing mouse testes while using an organ culture. We also investigated the difference between in vivo and ex vivo effects of NP exposure on testicular development.

## 2. Results

### 2.1. Development of the Mouse Fetal Testes in In Vitro Culture System

**Figure 1:**
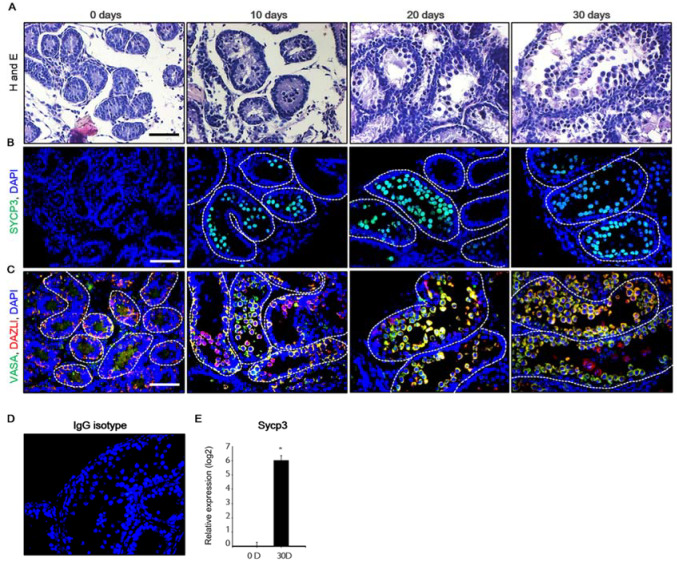
Development of mouse testicular fragments (MTFs) in the in vitro culture model. (**A**) Histological assessments performed using hematoxylin and eosin staining of MTFs cultured for 0, 10, 20, and 30 days. (**B**) SYCP3, (**C**) VASA, and DAZL proteins were detected in the MTFs after 0, 10, 20, and 30 days of culture using immunostaining. (**D**) The negative control stain using isotype-matched IgGs showed no specific signal. (**E**) The mRNA levels of the meiotic marker *Sycp3* in the MTFs were examined using quantitative polymerase chain reaction analysis. The relative quantification of mRNA is shown using the mean and standard error of the mean (*n* = 6) at log_2_ scale. * *p* < 0.05, Scale bars = 50 μm; each image was observed at the same magnification.

To investigate the effects of NP on neonatal testis development, mouse testicular fragments (MTFs) that were derived from 5.5-day-old testes were cultured. MTFs were observed from day 0 to day 30 following in vitro culture (Figure 1A. Immunostaining was then performed for common germ cell markers, including the meiotic markers Synaptonemal Complex Protein 3 (SYCP3), *Vasa*, and *Dazl* (Figure 1B,C), with IgG isotype being used as the negative control (Figure 1D). Normally, meiosis is initiated at eight days postpartum in neonatal mouse testes [29]. In this case, *Sycp3*-positive meiotic cells were observed in the inner seminiferous tubules from day 10 of the MTF culture. At day 0, *Vasa-* and *Dazl*-positive germ cells were both detectable in MTFs; however, these were absent from meiotic cells. Meiotic cells appeared in MTF after 10 and 20 days of culture, although spermatids were not observed. Lastly, the level of *Sycp3* transcripts in MTFs was significantly increased after 30 days of culture (Figure 1E). Together, these results demonstrate that spermatogonia developed into spermatocytes via meiosis within the MTF in vitro culture.

### 2.2. Effect of Nonylphenol on Germ Cells in MTFs

**Figure 2:**
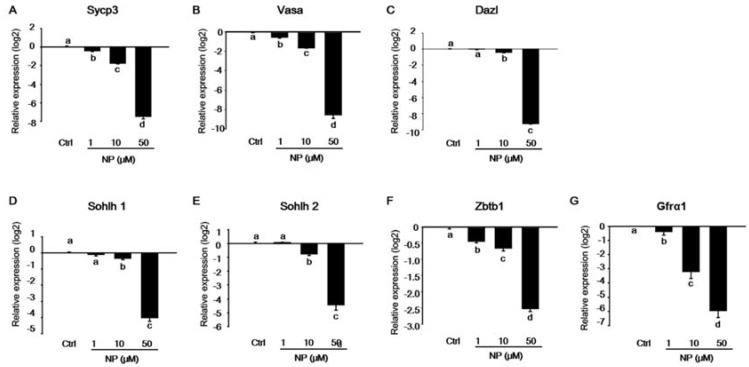
Expression of germ cell markers in 30-day cultured mouse testicular fragments (MTFs). The mRNA levels of the germ cell markers (**A**) *Sycp3*, (**B**) *Vasa*, (**C**) *Dazl,* (**D**) *Sohlh1,* (**E**) *Sohlh2*, (**F**) *Zbtb16*, and (**G**) *Gfra1* in the MTFs were determined using quantitative polymerase chain reaction. The relative quantification of mRNA is shown using the mean and the standard error of the mean (*n* = 6) at log_2_ scale. The levels of undifferentiated and differentiated germ cell markers distinctly decreased in a dose-dependent manner in 30-day cultured MTFs with nonylphenol (NP).

**Figure 3:**
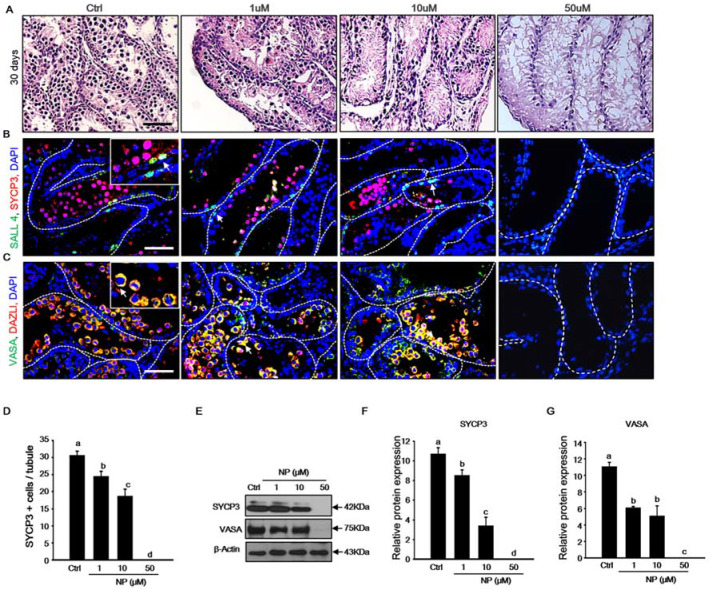
Toxic effect of nonylphenol (NP) on germ cell development. (**A**) Histological features of the mouse testicular fragments (MTFs) cultured for 30 days with 0, 1, 10, and 50 μM NP. (**B**) Meiotic and undifferentiated germ cells co-stained with SYCP3 and SALL4 antibody to confirm the occurrence of meiosis and the survival of undifferentiated germ cells in NP-exposed MTFs. SYCP3- and SALL4-positive cells (white arrow) were observed in 0, 1, and 10 μM NP-treated MTFs, but not in the 50 μM NP-treated MTFs. (**C**) MTFs co-stained with the germ cell markers VASA and DAZL in the presence and absence of NP (0, 1, 10, and 50 μM). The white arrow indicates VASA- and DAZL-positive cells in the germinal epithelium, and these cells were evident in 0, 1, and 10 μM NP-treated MTFs, but not in the 50 μM NP-treated MTFs. Scale bars = 50 µm. All images were acquired at the same magnification. (**D**) The average number of differentiated germ cells per seminiferous tubule was calculated on the basis of SYCP3 immunostaining in the 0, 1, and 10 μM NP-treated MTFs. At least 50 tubules were scored for each MTF (5–6 biological replicates). The data are shown as mean ± standard error. (**E**) The levels of SYCP3 and VASA proteins were measured in the MTF lysate with or without NP treatment, and β-actin was used as a loading control. The relative expression of (**F**) SYCP3 and (**G**) VASA in the MTF lysates is shown using the mean and the standard error of the mean (*n* = 5).

Our results showed that spermatogenesis partially progressed during the culture of MTFs *in vitro*. Therefore, we examined whether NP induced the inhibition of germ cell differentiation in the MTF culture. After exposure to NP, the expression of spermatogenesis-related genes, such as *Sycp3*, *Vasa*, *Dazl*, *Sohlh1*, and *Sohlh2,* decreased significantly in a dose-dependent manner as compared to that in the control (Figure 2A–E). In addition, there was a dramatic decrease in the expression of the undifferentiated germ cell marker genes, zinc finger and BTB domain containing 16 (*Zbtb16*), and GDNF family receptor alpha-1 (*Gfra1*), after exposure to 50 μM NP (Figure 2F,G). Histological changes in MTFs were observed following NP exposure, corresponding to quantitative PCR data. Notably, undifferentiated germ cells were located adjacent to the basement membrane of the seminiferous tubules, and spermatocytes were observed inside the spermatogonial cells in the control group. In contrast, in the NP-exposed MTFs, the spermatocytes gradually disappeared with an increase in the dose of NP (Figure 3A). Notably, cells were only present close to the basement membrane in MTFs that were exposed to the highest dose of NP (50 μM). We verified the presence of spermatocytes and spermatogonia in MTFs by immunostaining for markers of undifferentiated and differentiated germ cells. Undifferentiated and differentiated germ cell markers (*Sall4* and *Sycp3*) were both detectable in MTFs that were cultured with 0, 1, and 10 μM NP, but not 50 μM NP (Figure 3B). MTFs were also co-stained with antibodies against VASA and DAZL, which are germ cell markers. VASA- and DAZL-positive cells were both observed in 0, 1, and 10 μM NP-treated MTFs, but not in the 50 μM-treated group (Figure 3C). The number of SYCP3-positive cells was somewhat higher in the control MTFs than in the NP-exposed MTFs (Figure 3D).

In addition, we measured the protein expression of SYCP3 and VASA by immunoblotting. Although both of the proteins were detected in the control and MTFs that were treated with 1 and 10 μM NP, they were undetectable in MTFs treated with 50 μM NP (Figure 3E). Quantification by densitometry showed that the protein expression of SYCP3 and VASA was significantly reduced (Figure 3F,G). Together, these data indicate that NP significantly reduced the number of germ cells and inhibited germ cell differentiation in cultured MTFs.

### 2.3. Effect of Nonylphenol on Sertoli Cells in MTF Culture

**Figure 4:**
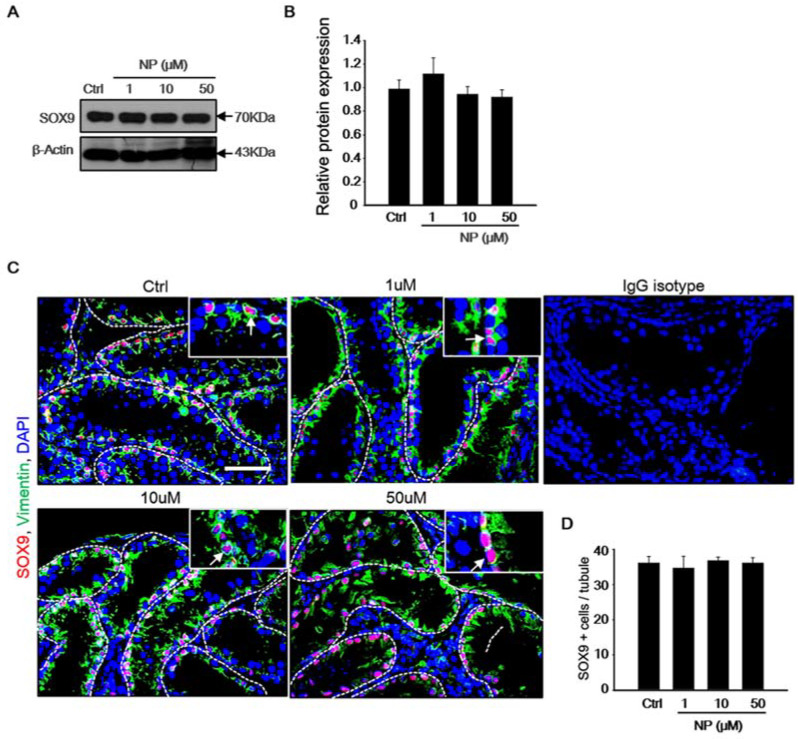
Effect of nonylphenol (NP) on Sertoli cells during mouse testicular fraction (MTF) culture. (**A**) Representative immunoblots of SOX9, a Sertoli cell-specific marker, in 30-day cultured MTFs with or without NP. β-actin was used as the loading control. (**B**) Ratios of SOX9 to β-actin are shown as the mean and the standard error of the mean (*n* = 5). No significant difference was noted between the 1, 10, and 50 µM NP-treated MTFs and controls. (**C**) Double immunohistochemistry of the MTF sections was used to examine the localization of SOX9 and vimentin in MTFs cultured for 30 days with NP and with the IgG isotype-negative control. Scale bars = 50 µm. All of the images were acquired at the same magnification. (**D**) The average number of SOX9-positive Sertoli cells in the tubules was calculated on the basis of immunostaining. At least 50 tubules were scored for each MTF (5–6 biological replicates). The expression of SOX9 and number of SOX9-positive cells in each tubule did not differ significantly between the NP-treated and untreated MTFs. The data are presented as mean ± standard error.

The expression of the Sertoli cell-specific marker, SOX9, was observed using immunoblotting to investigate whether exposure of MTF culture to NP had an effect on Sertoli cells (Figure 4A). The protein levels of SOX9 did not change in NP-treated MTFs after 30 days of culture. Quantification by densitometry showed that the level of SOX9 did not change significantly in the NP-treated samples (Figure 4B). Additionally, immunostaining for SOX9 and vimentin was performed in order to confirm the effect of NP on Sertoli cells (Figure 4C). SOX9-positive Sertoli cells were located near the basement membrane (Figure 4C, white arrow). Additionally, the number of Sertoli cells in the control and treated MTFs was compared (Figure 4D). IgG isotype control was used as the negative control for the staining. The number of Sertoli cells was not significantly different between the control and NP-exposed MTFs (1–50 μM). This indicated that the Sertoli cell number was not affected by NP treatment in cultured MTFs.

### 2.4. Effect of Nonylphenol on mRNA Expression of Steroidogenic Genes in MTFs

**Figure 5:**
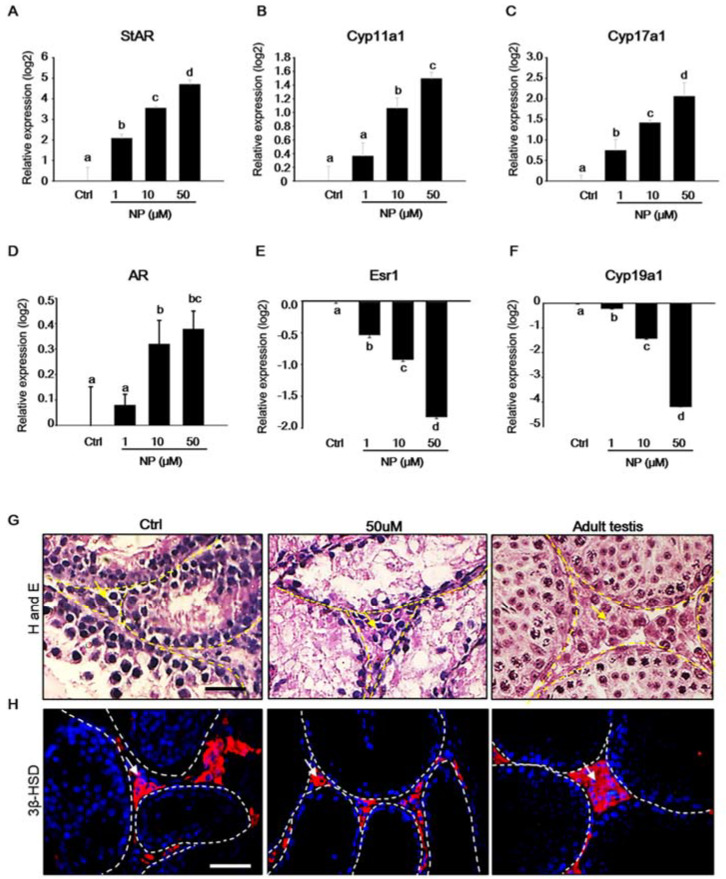
Expression of Leydig cell markers in the mouse testicular fragments (MTFs) after nonylphenol (NP) treatment. MTFs were cultured in the absence and presence of NP (1–50 μM) for 30 days. (**A**) *Star*, (**B**) *Cyp11α1*, (**C**) *Cyp17a1*, (**D**) *Ar*, (**E**) *Esr1*, and (**F**) *Cyp19α1* mRNA levels in the MTFs were determined using quantitative polymerase chain reaction. The relative quantification of mRNA is shown as the mean and the standard error of the mean (*n* = 6) at log_2_ scale. The expression of steroidogenesis-related Leydig cell markers such as *Star, Cyp11α1,* and *Cyp17a1* increased dose-dependently in the NP-treated MTFs. (**G**) The histological image shows that Leydig cells are located in the interstitial region in NP-treated (50 μM) and untreated MTFs (indicated by yellow arrow), and adult testes were used as positive controls. Moreover, (**H**) 3β-HSD protein was detected in MTFs with and without NP treatment by immunostaining (indicated by white arrow). Scale bars = 50 µm. All of the images were acquired at the same magnification.

Steroid hormones are activated during testicular development and they are critical for spermatogenesis. In particular, androgen, a steroid hormone that is secreted by Leydig cells in the testis, is essential for normal testis development [30,31]. As steroidogenic enzymes are involved in steroid hormone biosynthesis, the expression of steroidogenic enzymes in cultured MTFs was investigated in order to evaluate the effect of NP on steroidogenesis. Studies have reported that NP affects steroidogenesis in Leydig cells [32,33]. Our results show that, when compared to that in the control, the mRNA expression of the steroidogenic acute regulatory gene (*Star*), *Cyp11α1, Cyp17α1*, and androgen receptor (*Ar*) increased with increasing concentrations of NP (Figure 5A–D). In contrast, the expression of estrogen receptor 1 (*ESR1*) and *Cyp19α1* decreased in NP-treated MTFs in a dose-dependent manner (Figure 5E,F). In addition, the histological observation of MTFs revealed that Leydig cells were present in the interstitial regions, irrespective of the presence or absence of NP (Figure 5G). MTFs were stained for 3β-hydroxysteroid dehydrogenase (3β-HSD), a Leydig cell-specific marker [34]. *3β-HSD*-positive Leydig cells were detected in both NP-treated and untreated MTFs, similarly to that in stained adult testis used as a positive control (Figure 5H).

## 3. Discussion

The neonatal stage is an important developmental period that is extremely sensitive to chemical exposure [35,36]. It is easier to evaluate the impact of chemicals in adult animal models than in neonatal animals. Therefore, this study aimed to determine the reproductive toxicity of NP in neonatal testes while using the organ culture method. We found that the exposure of MTFs to NP resulted in the destruction of testicular germ cells, but did not affect the Sertoli and Leydig cells. Notably, a high dose of NP (50 μM) completely inhibited spermatogenesis in MTFs via germ cell depletion. However, germ cells were still observed in MTF organ cultures that were exposed to low doses of NP (1 and 10 μM).

Several studies reported that 1–200 nM NP was detected in human blood and urine [7,8,9,10], but the NP dose in our experiment is higher than the physiological levels in humans. In rodents, another researcher administered 21–188 mM NP for an in vivo experiment [37].

Other researchers have demonstrated that NP exhibits adverse effects on the male reproductive system. The exposure to NP in neonatal male pups resulted in decreased testis, epididymis, seminal vesicle, and ventral prostate sizes and increased frequency of cryptorchidism [38]. Duan et al. reported that NP disrupts spermatogenesis and results in spermatotoxic effects, such as fructose depletion, oxidative stress, hormone deficiency, and testicular cell apoptosis [39].

Although several studies have reported that NP has the potential to induce cytotoxicity and apoptosis in Sertoli and Leydig cells [32,40,41], our results suggest that only NP destroyed germ cells at a concentration of 50 μM, while no effect was observed on the number of Sertoli cells. In addition, germ cells were observed in NP-treated MTFs, but not in those that were treated with a high dose of NP (50 μM). Other groups demonstrated that NP induced apoptosis in mouse TM4 Sertoli cells via reactive oxygen species generation and extracellular signal-regulated kinase activation [40]. Moreover, Duan et al. reported that high doses of NP induced apoptosis via the generation of oxidative stress in the testes [22]. Similarly, the expression of oxidative stress-related genes, including superoxide dismutase 1 (*Sod1*), catalase (*Cat*), glutathione peroxidase (*Gpx*), and Bcl-2 related ovarian killer (*Bok*), was significantly increased in 50 μM NP-exposed MTFs as compared to that in the control. A similar pattern was observed for the expression of apoptotic genes, such as *Fas* and TNF receptor-associated factor (*Traf3*) (Supplementary Figure S1A). These results suggest that in vitro MTF cultures may serve as a model system for assessing testis toxicity.

Interestingly, the level of both Wilms’ tumor suppressor gene 1 (*Wt1*) and anti-Mullerian Hormone (AMH) transcripts was higher in the NP-exposed MTFs than in the control in spite of the unaltered number of Sertoli cells (Supplementary Figure S1B). Chen et al. reported that the cross-talk between Sertoli and Leydig cells is important for steroidogenesis. Wt1 is crucial for adult Leydig cell morphology in the testes during postnatal development, as the loss of Wt1 leads to the downregulation of paracrine factor, which in turn causes a decrease in steroidogenic enzyme expression and reduced testosterone production in Leydig cells [42]. Meanwhile, herein, we observed increased expression of both *Wt1* and steroidogenic genes in NP-treated MTFs. Moreover, AMH, a marker of immature Sertoli cells, was highly expressed in five-day testes, while decreased *Amh* expression in 10-day testes could be attributed to the maturation of Sertoli cells in rodents [43]. Although the underlying cause is unclear, the population of immature Sertoli cells might have been enriched in NP-exposed MTFs as compared to that in the controls. Further, this study investigated the effect of NP on steroidogenesis in Leydig cells. Our results demonstrate that NP increases the expression of steroidogenesis-related genes, such as *Star*, *Cyp11a1,* and *Cyp17a1*, in MTFs, which is supported by other studies. It has been reported that NP stimulates steroidogenesis at a concentration of 127.5 µM by increasing the activity of P450SCC and stimulates the expression of STAR protein in primary Leydig cells in rats [33]. Moreover, androstenedione production increases with rising NP concentration (1, 2.5, and 5 μg/mL) in mouse Leydig cells [44]. In an in vivo study, the plasma testosterone level was significantly higher in NP-treated rats than in the control rats after a seven-day exposure to NP [45]. In our study, the expression of Ar was significantly higher in MTFs that were treated with 10 and 50 μM NP than in the control. However, other studies have shown contradictory results; some found that high doses of NP inhibit testosterone secretion by Leydig cells and induce their death *in vitro,* while low doses of NP have been show to increase testosterone synthesis [46]. In addition, Kazemi et al. showed that NP downregulated the level of testosterone in rat blood samples [47]. It is possible that the biological action of NP differs between in vitro and in vivo systems as the endocrine system is a complex network of glands and organs, which is difficult to replicate *in vitro*. NP is an endocrine-disrupting chemical (EDC) with estrogenic activity. Bartke et al. reported that treatment with estradiol in vivo decreases testosterone production via direct inhibitory effects on testicular steroidogenesis; meanwhile, when decapsulated mouse testes were treated with estradiol in the absence of hCG, testosterone accumulation was not inhibited [48]. It is well known that the enzyme Cyp19a1 converts testosterone into estrogen and regulates the concentration of sex steroidal hormones [49,50]. EDCs can induce estrogen-like or androgen-like effects by binding to hormone receptors, including the estrogen receptor (ER) and androgen receptor (AR). Interestingly, in this study, while the expression of AR was increased, the expression of *Esr1* and *Cyp19a1* mRNA was significantly decreased after exposure to NP. This could be because exposure to high concentrations of NP induced androgen synthesis via the over-expression of *Star, Cyp11a1, Cyp17a1*, and *Ar*. Simultaneously, NP inhibits Cyp19a1-mediated androgen-to-estrogen conversion, although the hormone levels were not measured in MTFs in the in vitro models [50,51].

Few studies have reported a relationship between NP and Cyp19a1, and most of these studies were conducted in the fish models. NP acted as an agonist of ER in fish [51] and showed a strong estrogen effect on sex differentiation, leading to a change in sex ratio in the F1 generation. An upregulation of both Cyp19a1 and ER α was observed in the NP-exposed group at 20 days post-fertilization in the F1 generation in zebrafish [52], whereas exposure to 100 and 1000 nM NP significantly suppressed Cyp19a1 expression in rare minnow juveniles [53]. NP was also shown to induce testicular abnormality by disturbing E2 metabolism in *Zoarces viviparus* [54].

In mammals, NP enhanced ER α activity by recruiting it to the target gene promotor in human neuroblastoma [55]. Decreased mRNA expression of *Ers1, Ers2, Ar*, and progesterone receptor was observed in the mouse TM4 Sertoli cells that were exposed to 4-(1-ethyl-1-methylhexyl)phenol (4-NP65), the main isomer in technical NP mixtures [56]. However, the detailed molecular mechanism underlying the relationship between NP and Cyp19a1 in rodent testes is still unclear.

In summary, in this study, we estimated the toxicity of NP in developing mouse testes while using an organ culture method for the first time. Exposure of MTFs to a high dose of NP (50 µM) led to specific depletion of germ cells in the seminiferous epithelium in MTFs, but not Leydig or Sertoli cells, resulting in spermatogenic failure. This study provides a foundation for the use of organ culture methods for assessing the reproductive toxicity of NP, thus eliminating the requirement for animal models. However, the molecular mechanisms underlying NP-induced germ cell depletion still need to be fully evaluated.

## 4. Materials and Methods

### 4.1. Animals

CD-1 female mice and two-day-old male pups were obtained from Orient Bio (Seoul, Korea) and then maintained for a few days. Five-day-old male pups were used for the organ culture experiment. The mice were housed with a 12 h light: 12 h dark photoperiod, and temperature was maintained at 21 ± 1 °C. Konkuk University approved the study protocol. The Institutional Animal Care and Use Committee (IACUC) of Konkuk University granted ethics approval for the project (Protocol KU19132, 1 August 2019).

### 4.2. Organ Culture Method and Test Compound

MTFs from five-day postpartum neonates were cultured, as described in other previous studies [23,25]. First, neonatal testes were dissected and encapsulated in α-minimum essential medium (α-MEM: Welgene, Daegu, Korea). Encapsulated testes were carefully dissected by forceps into six to eight pieces of approximately 2 mm in diameter, and four to five tissue fragments were transferred onto 1.2% (*w/v*) agarose gel (Sigma–Aldrich, St. Louis, MO, USA) containing 1 mL of α-MEM (Welgene) and 10% (*v/v*) Knock-out Serum Replacement (Thermo Fisher Scientific, Walthan, MA, USA). Three to four agarose gels were placed in each well of a six-well plate (Corning Inc., Corning, NY, USA). MTFs were incubated under conditions of 34 °C and 5% CO_2_, and the medium was changed twice a week for 30 days. NP (Sigma–Aldrich, St. Louis, MO, USA) was dissolved in DMSO and then diluted in the culture medium to the final concentrations of 1, 10, and 50 μM. The concentration of NP was chosen based on other studies [57,58]. The 30-day MTF cultures were analyzed to confirm whether more than 60% of the seminiferous tubules contained differentiated germ cells for toxicity testing in accordance with other studies [59].

### 4.3. Histological Analysis and Immunostaining

Cultured MTF tissues were fixed in 4% paraformaldehyde at 4 °C overnight and then gradually dehydrated while using 100% ethanol for 60 min. After dehydration, the tissues were cleaned twice with xylene for 60 min. each, embedded in paraffin, sectioned at a thickness of 5 µm using a microtome (Leica, Nussloch, Germany), and then placed on glass slides. The method for tissue staining has been described previously [26]. Tissue sections were processed for hematoxylin and eosin staining after deparaffinization. MTF sections, from five different areas of the testis, for each biological replicate, were analyzed, and a blinded analyst examined stained samples using a microscope. For immunohistochemistry, the sections were rehydrated using xylene and 90–100% ethanol. Antigen retrieval was performed in 10 mM sodium citrate buffer, and the samples were heated to near-boiling temperature for 15 min. The tissue was then permeabilized in phosphate-buffered saline (PBS), containing 0.02% Triton X-100, for 10 min. at 25 °C, and then transferred into blocking solution (1% bovine serum albumin in PBS) for 30 min. The tissues were then incubated with primary antibody diluted in 1% BSA in PBS at 4 °C overnight. Following three washes in PBS (20 min. each), the tissue was incubated with secondary antibodies, diluted 1:300 in 1% BSA in PBS, for 1 h at 25 °C. Alexa Fluor 488 goat anti-mouse IgG, Alexa Fluor 568 donkey anti-rabbit IgG, Alexa Fluor 568 donkey anti-mouse IgG, and Alexa Fluor 488 goat anti-rabbit IgG from Life Technologies were used as the secondary antibodies. Table 1 provides details regarding the antibodies used for immunostaining. The tissues were then washed several times with PBS and incubated with 1 µg/mL 6-diamidino-2-phenylindole (DAPI) in PBS for 5 min., and coverslips were placed along with mounting solution (DAKO; Carpinteria, CA, USA; S3025). The samples were observed while using a fluorescence microscope (Nikon, Tokyo, Japan). All of the images were captured using a Nikon E-800 microscope with Motic Image Advanced 3.2 Software (Kowloon, Hong Kong). For quantitative analysis, more than five different MTFs from each experimental group were analyzed. Testicular cross-sections with at least 60 tubules from 5–6 sections were scored for each MTF.

### 4.4. RNA Isolation and Quantitative PCR

The total RNA from MTFs was extracted using a Qiagen RNeasy Mini Kit (Qiagen, Cat: 74106) with on-column DNase treatment (Qiagen, Cat: 79254), according to the manufacturer’s instructions. Reverse transcription was performed using SuperScript III Reverse Transcriptase (Invitrogen, CA, USA) with Oligo(dT30)-primer and 1 µg RNA, following the manufacturer’s instructions. Quantitative PCR was performed, as described previously [26], while using a real-time PCR system (Qiagen, Rotor-Gene Q). Table 2 lists the primers used in this study. Denaturation and polymerase activation were performed at 94 °C for 1 min., followed by 40 cycles of 94 °C for 10 s, 57 °C for 10 s, and 72 °C for 20 s. The data were analyzed using the Ct method [60], and *Gapdh* was used as the control gene. After normalization to *Gapdh* cDNA levels, which were reflected in the ΔCt values, the relative quantification (RQ) of the fold change for each treatment as compared with that of the reference control was determined using Equation (1). The RQ mean and SEM were plotted on a log_2_ scale.

(1) $$\mathrm{RQ}=\frac{2\left(-\Delta Ct\right)}{2\left(-\Delta Ct\;reference\right)}$$

### 4.5. Immunoblotting

Proteins from cultured MTFs were extracted using ice-cold RIPA buffer (Thermo Fisher Scientific, MA, USA) containing protease inhibitors by vortexing for 30 min. The protein samples were quantified while using the BCA Protein Assay kit (Pierce Biotechnology, Rockford, IL, USA; #23 227). Samples containing equal concentrations of protein were loaded into wells containing 4–20% mini-TGX gels (Bio-Rad, Hercules, CA, USA; #456–1096), and electrophoresis was performed. Subsequently, the samples were transferred onto polyvinylidene difluoride membranes. The blots were probed overnight at 4 °C with a primary antibody diluted in TBST and 1% BSA. Table 1 lists the primary antibodies used in the study. Following three washes in TBST (30 min. each), the blots were incubated for 1 h with 1:2000 dilutions of anti-mouse and anti-rabbit IgG and HRP-linked antibodies (Santa Cruz Biotechnology, 1:1000) prepared in TBST and 1% BSA. The ECL Western Blotting Substrate (Thermo Scientific, Rockford, IL, USA; #32106) and HyBlot CL autoradiography film (Denville Scientific, Metuchen, NJ, USA; #E3018) were used for signal testing. β-actin was used as the control for normalization.

### 4.6. Statistical Analysis

The SPSS statistical package ver. 15.0 for Windows (IBM Corp., Somers, NY, USA) was used for data analysis. All of the data are expressed as mean ± standard error. The differences among three or more groups were evaluated by one-way ANOVA, followed by Tukey’s honest significant difference test. The differences between two groups were evaluated by Student’s *t*-test.

## Figures and Tables

**Table 1:** List of antibodies used for immunostaining.

Antibody	Company	Catalog Number	Dilution
VASA	Abcam	ab13840	1:300
SALL4	Abcam	ab57577	1:300
Sox9	Abcam	Ab185230	1:200
Vimentin	Santa Cruz Biotech	sc-373717	1:300
SYCP3	Abcam	ab15093	1:300
DAZL	BIO-RAD	MCA2336	1:200
3βHSD	Santa Cruz Biotech	sc30820	1:200
βActin	Santa Cruz Biotech	ab13840	1:1000

**Table 2:** Primers used for reverse transcription-polymerase chain reaction using mouse cDNA.

Gene	Forward Primer	Reverse Primer
*Sycp3*	5′-CAGATGCTTCGAGGGTGTG-3′	5′-AAGGTGGCTTCCCAGATTTC-3′
*Dazl*	5′-GTCGAAGGGCTATGGATTTG-3′	5′-ACGTGGCTGCACATGATAAG-3
*Vasa*	5′-CCGCATGGCTAGAAGAGATT-3	5′-TTCCTCGTGTCAACAGATGC-3
*Zbtb16*	5′-CCACCTTCGCTCACATACAG-3′	5′-TTGCCACAGCCATTACACTC-3′
*Sohlh1*	5′-CATCTGCTGTGTCTCGGGTA-3′	5′-GCTGGAAGACTCTGGCTCAC-3′
*Sohlh2*	5′-TGAGACGAGAACGCATCAAG-3′	5′-CCTCTGTGATGTGGCTGAGA-3′
*AR*	5′-GGCGGTCCTTCACTAATGTC-3′	5′-GACAGGTGCCTCATCCTCAC -3′
*ERα*	5′-GCACAAGCGTCAGAGAGATG-3′	5′-AGGACAAGGCAGGGCTATTC-3′
*Cyp19* *α1*	5′-TTGAGACGATTCCAGGTGAAG-3′	5′-ATTTCCACAAGGTGCCTGTC-3′
*Cyp17* *α1*	5′-TCCAGCATTGGAGAGTTTGC-3′	5′-ATGAGATGGCTTCCTGTTGG-3′
*Cyp11* *α1*	5′-GACAATGGTTGGCTAAACCTG-3′	5′-GGGTCCACGATGTAAACTGAC-3′
*Star*	5′-TGGGCATACTCAACAACCAG-3′	5′-GTCTACCACCACCTCCAAGC-3′
*Sod1*	5′-GGGTTCCACGTCCATCAGTA-3′	5′-AGTCACATTGCCCAGGTCTC-3′
*Cat*	5′-GCAGATACCTGTGAACTGTC-3′	5′-GTAGAATGTCCGCACCTGAG-3′
*Gpx*	5′-TTCGGACACCAGGAGAATGG-3′	5′-TAAAGAGCGGGTGAGCCTTC-3
*Bax*	5′-GCTGACATGTTTGCTGATGG-3′	5′-GATCAGCTCGGGCACTTTAG-3′
*Bok*	5′-CTGCCCCTGGAGGACGCTTG-3′	5′-CCGTCACCACAGGCTCCGAC-3′
*Fas*	5′-CTGATCCTCATTCCCGTACC-3′	5′-ATCATTGGCACCTCTTCAGC -3′
*Traf3*	5′-AGAGTGAGTTGAGTGCACACTT-3′	5′-TACCGCGGAGCTGGCCTCAT-3′
*Wt1*	5′-ATCCCAGGCAGGAAAGTGTG-3′	5′-GTGCTGTCTTGGAAGTCGGA-3′
*Amh*	5′-CCTGGAGGAAGTGACATGG-3′	5′-CAGGGTAGAGCACCAGCAG-3′

## Supplementary Materials

The following are available online at https://www.mdpi.com/1422-0067/21/10/3491/s1, Figure S1: The gene expression of ROS, apoptotic and sertoli cell marker.
