# Supplementary material for: Toxic Effects of Nonylphenol on Neonatal Testicular Development in Mouse Organ Culture
Source: Int J Mol Sci. 2020 May 15;21(10):3491. doi: 10.3390/ijms21103491 (PMC7279013; doi:10.3390/ijms21103491)
Supplement: Supplementary file 1 [file ijms-21-03491-s001.pdf]

Toxic Effects of Nonylphenol on Neonatal Testicular Development

Hyun-Jung Park [1], Mingtian Zhang [1], Won-Young Lee [2], Kwon-Ho Hong [1], Jeong Tae Do [1], Chankyu Park [1] and Hyuk Song [1*]

[1] Department of Stem Cell and Regenerative Biology, Konkuk University, 1 Hwayang-dong, Gwangjin-gu, Seoul 05029, Republic of Korea

[2] Department of Beef Science, Korea National College of Agricultures and Fisheries, Jeonju-si, Jeonbuk, 54874, Republic of Korea

* Correspondence: songh@konkuk.ac.kr; Tel.: (+82-43-840-3522)

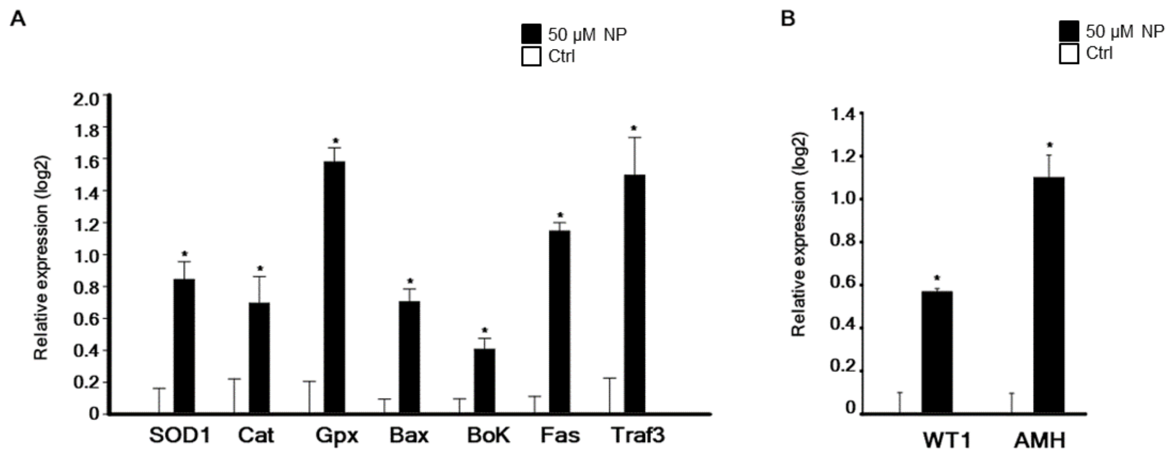

Supplementary Figure 1

(A) The expression of oxidative stress-related genes, *Sod1, Cat*, *Gpx*, *Bax*, and *Bok*, and apoptotic genes, *Fas* and *Traf3*. (B) Sertoli cell specific genes, *Wt1* and *Amh* in the absence and presence of NP (50 μM) after 30 day MTF *in vitro* culture. *GAPDH* was used as the control gene Relative quantification of mRNAs is shown using the mean and standard error of the mean (n = 6). *P < 0.05.
